# Cross-cultural adaptation and validation of Pamela Reed’s Self-Transcendence Scale for the Spanish context*

**DOI:** 10.1590/1518-8345.2750.3058

**Published:** 2018-12-10

**Authors:** Alberto Pena-Gayo, Víctor Manuel González-Chordá, Águeda Cervera-Gasch, Desirée Mena-Tudela

**Affiliations:** 1 Universitat Jaume I, Facultad de Ciencias de la Salud, Castellón, Comunidad Valenciana, Spain.

**Keywords:** Psychological Adaptation, Self-Transcendence, Holistic Nursing, Spanish, Spirituality, Validation Studies

## Abstract

**Objectives:**

the current study aimed to adapt the Self-Transcendence Scale (STS) to the Spanish context and analyse its psychometric properties.

**Method:**

the STS was administered to a general Spanish population of adults (i.e., older than 20 years; n = 116) through an online platform. The Psychological Well-Being (PWB) and the Functional Assessment of Chronic Illness Therapy - Spiritual Well-being - modified version for healthy people (FACIT-Sp-Non-Illness) scales were also applied in two moments separated by an interval of 15 days.

**Results:**

the results of the validation included the following statistics: α_*t*_ = 0.772 (test) and α_*rt*_ = 0.833 (retest); ICC = 0.278 (*p* = 0.097, intraclass) and 0.932 (*p* < 0.001, interclass); a Bland-Altman confirmation of the test/re-test (TRT) concordance; global content validity coefficient (S-CVI) = 0.92; *r*_1_ = 0.636 (PWB) and *r*_2_ = 0.687 (FACIT-Non-Illness; both *p* < 0.001); and three factors explained 42.3% of the variance. The STS showed positive apparent validity and feasibility.

**Conclusions:**

the Spanish version of the STS is valid for use in the general population, with updates relative to the Colombian version that include more natural wordings, syntactic corrections, inclusive language, a better definition of the concepts, and an alternative factor model.

## Introduction

Throughout the life cycle, humans experience circumstances that can overwhelm their coping resources, thereby establishing a dynamic process of adaptation that will bring about a new state of maturity through a personal transformation. Through this process, the concept of self-transcendence emerges, which is understood as the relationship between the personality and spiritual behaviours of an individual; this concept is associated with creativity, imagination, and the ability to accept uncertainty. The term is also related to vulnerability, a concept that alludes to the awareness of a person about his or her mortality.

In the field of nursing, Pamela Reed has discussed this topic in depth in her theory of self-transcendence^1^ ^-^ ^2^, which was developed from the conceptual model of Martha Rogers. Reed relates self-transcendence to vulnerability and well-being. Vulnerability induces greater self-transcendence and, in turn, greater well-being. Each of these three concepts is regulated by personal and contextual mediating factors, which is where nursing should apply. Reed defines self-transcendence as an individual’s ability to expand his or her own limits in the following dimensions: interpersonal (in relation to others), intrapersonal (in relation to oneself), transpersonal (in relation to a spiritual dimension), and temporal (by the integration of the past and future to give meaning to the present).

Reed presents self-transcendence as an evolutionary capacity that provides purpose and meaning to human existence in the face of individual and environmental limits, which can be evaluated at a specific moment in the life cycle.

The Self-Transcendence Scale (STS) was developed based on the Developmental Resources of Later Adulthood (DRLA), which observed that a single factor explained 45.2% of the variance. Its content validity was confirmed based on a contrast with the literature concerning the conceptualisation of the life cycle of human development and various studies conducted with older adults. In the original version, a Cronbach’s alpha (α) of 0.8 was obtained, with variations in later studies. Its construct validity is demonstrated via analyses of convergence (well-being) and divergence (depression).

The STS is currently a consolidated scale that has been translated into different languages as Korean, Swedish, Persian or Norwegian^3^ ^-^ ^6^; however, this scale has not been adapted to the Spanish context. One reference exists regarding a version for adolescents adapted to the Colombian context, although the articles that have cited this paper are unpublished manuscripts^7^ ^-^ ^8^. A cross-cultural adaptation and validation in the Colombian context was found^9^, and this reference is the only one in the Spanish language, showing an internal validity of α = 0.85.

Self-transcendence theory favours a humanistic approach to nursing that starts with prioritising a set of technical skills and moves to others that promote an internal process that exists within and between complex human systems. Its use in Spain might spur the beginning of new investigations that complement this view. For all these reasons, a cross-cultural adaptation of the scale and its validation for future studies related to this subject was considered pertinent.

The general objective of this study was to adapt and validate the STS to the Spanish context. The specific objectives were as follows: (a) translate and culturally adapt the STS via the translation/back-translation method; (b) analyse the apparent and content validity through consolidation via a panel of experts; and (c) conduct a pilot study to analyse the psychometric properties of validity and reliability.

## Methodology

A descriptive and cross-sectional observational study for instrument validation was conducted between November 2016 and September 2017. The following steps were followed: (i) cross-cultural adaptation; (ii) content validity analysis; (iii) feasibility and psychometric property calculation.

Two native Spanish-speaking translators participated in the direct translation, and another two native English-speaking translators participated in the back-translation. A fifth translator was reserved for possible disagreements, selected using the same academic criteria. The translators worked independently and were presented with the original document for translation following the guidelines of the International Test Commission (ITC). The results were passed on for a blind peer review with the following precepts: (a) maximum fidelity to the original scale; (b) Spanish cultural context; (c) generic target population; (d) understandable by a 12-year-old student^10^. The chatstep.com platform was used to discuss differences and reach agreement. Throughout the process, the methodological recommendations regarding the cross-cultural adaptation of evaluation scales were followed^10^ ^-^ ^12^.

The content validity of the STS was examined by a group of 20 experts. The inclusion criteria for participation on this panel were university graduates in nursing or psychology, experts in research, and native Spanish speakers. The experts received the questionnaire via e-mail and (a) assessed the conceptual equivalence between the translated version and the original version (yes/no), (b) assessed the relevance of each item using a four-point Likert scale (where 1 represents “irrelevant” and 4 represents “highly relevant”); and (c) provided suggestions and comments. A content validity analysis was performed via the content validity index (I-CVI, where adequate validity ≥ 0.8 for each item) and the global content validity coefficient (S-CVI, where adequate validity ≥ 0.8 for the complete questionnaire)^13^ ^-^ ^14^.

Finally, the feasibility and psychometric properties of the questionnaire were studied. The scale was administered to a sample of volunteers who were 20 years old or above. This criterion was verified during the administration of the scale, which was conducted through the application onlineencuesta.com, a previous dissemination via social networks and national nursing schools, and via promotional posters at the university, and health and social centres of Alcalá de Henares in Madrid, Spain. Two criteria were followed regarding the sample size: a minimum of 50 cases or 5-10 individuals per item^15^, which indicated a minimum of 75 cases (15 items).

The questionnaire battery included the STS (a 15-item one-dimensional scale that measures the degree of self-transcendence, scored with an ascending four-point Likert scale with a score that ranges between 15 and 60 points), the Psychological Well-Being (PWB) Scale (a 29-item, six-dimensional instrument assessed with an ascending six-point Likert scale, with an internal consistency of α = 0.84 [Spanish version])^16^ and the Functional Assessment of Chronic Illness Therapy, Spiritual Well-being, modified version for healthy people (FACIT-Sp-Non-Illness) Scale (a 12-item, three-dimensional instrument assessed with a five-point Likert scale, with an internal consistency of α = 0.87 [original version])^17^. In addition, sociodemographic variables (age, sex, marital status, employment status, educational level, number of children, and autonomous community) were collected, as were control variables that recorded the presence or absence of chronic pathology, perceived health status, recent hospitalisation, and current level of concern.

Feasibility was studied based on the comments of the experts and participants regarding the scale, time of completion, and number of missing scores. Reliability was analysed using the intraclass correlation coefficient (ICC), where an adequate value is ≥ 0.70. The inter-observer (where interclass considers the participants as observers and the items as the valuation objects) and intra-observer (where intraclass considers the researcher as the observer and the scores as the valuation object at two different moments) reliabilities were analysed, so that the participants received a mailing days after the completion of a new link to the questionnaire, which this time included a control variable that determined whether the participant had experienced major life changes during that period. Student’s t-test for paired samples, the Bland-Altman plot^18^ (which represents the average of each pair of test and retest values on a horizontal axis and the difference of each pair of values on a vertical axis), and the Kaplan-Meier graph^18^ (which represents the absolute difference between pairs of measurements on a horizontal axis and the proportion [i.e., accumulated number] of cases that are at least equal to each of the observed differences on the vertical axis) were applied to conduct the analysis. The test-retest (TRT) interval should be adequate to avoid bias because of changes in the studied phenomenon (long term) or based on recall of the test responses (short term)^19^. An interval of 15 days was considered adequate.

Criterion validity was determined based on concurrent validity with the PWB and FACIT-Sp-Non-Illness scales. The correlation was examined with Pearson’s *r* after standardising the scores in the form of a ratio (i.e., the score obtained divided by the maximum possible score) that was ordered to match the TRT scores of each participant. The construct validity was examined using an exploratory factorial analysis (EFA) and confirmed by a confirmatory factor analysis (CFA). The following goodness-of-fit indices were calculated^6^: (a) chi-square (χ^*2*^ ), where smaller scores denote better fits; (b) root mean square error of approximation (RMSEA), with values < 0.05 indicating a good fit; (c) standardised root mean square residual (SRMR), with values < 0.05 indicating a good fit; (d) comparative fit index (CFI), with values ≥ 0.97 indicating a good fit; (e) normed fit index (NFI) and non-normed fit index (NNFI), with values ≥ 0.90 and ≥ 0.95 indicating a good fit, respectively; and (f) goodness-of-fit index (GFI), with a recommended value of ≥ 0.90 and adjusted GFI (AGFI) with ≥ 0.85 showing a good fit. As a criterion for the relevance of a factorial analysis^20^, Bartlett’s sphericity test (according to a *p*-value) and the Kaiser-Meyer-Olkin test (significant if KMO> 0.6) were performed. Internal consistency was studied using Cronbach’s alpha coefficient (α ≥ 0.70).

The statistical analyses were executed with the statistical packages “R Commander”, “irr”, “psych”, “RCmdrPlugin.Survival”, and “RCmdrPlugin.FactoMineR”within R, version 3.4.1. The Bland-Altman plot was constructed using Epidat, version 4.2. A level of significance of p ≤ 0.05 was established.

Following current legislation on human research, participant permission was requested through informed consent, which was inserted into the online platform. A logical sequence was programmed to continue only when the participants read the conditions and provided consent; otherwise, the user was automatically redirected out of the questionnaire, thereby ending the intervention. In addition, the Deontological Commission of Jaume I University provided a favourable report of the current investigation. In addition, permission was requested from all of the authors of the scales used. In accordance with Spanish legislation regarding the protection of personal data, a file was registered for this study with the possibility of access, modification, or cancellation of the data by the participants. The data were archived and guarded by the principal investigator, encrypted in a *.zip* file. The authors have no conflicts of interest to report.

## Results

Two native Spanish-speaking translators translated the scale after agreeing to the following guidelines: (a) a present indicative verb should be used in place of a gerund, (b) treatment of courtesy should replace informalism, (c) inclusive language should be used, (d) the original item valuation scale should be respected, and (e) specific modifications should be made under the “translation is not an exact science” premise (a literal note from the debate among the translators). A verb in the present tense “interprets the receiver in an operative way for this type of text” (literal note). In the reverse translation, it was necessary to use the fifth translator. The following pairs were specified: (a) item 9, yearning/keen; (b) item 10, move on/succeed; (c) item 12, meaningful/make sense; (d) item 13, when necessary/if I were unable; and (e) item 15, old baggage/past worries. The author of the scale was contacted, who validated all translations except for item 10.

Of the 20 experts who agreed to collaborate, one decided not to assess the relevance of the items after not accepting the term “expert”; as such, this person only participated in the conceptual equivalence session, in which only items 10 and 15 scored low (0.750 and 0.736). Based on the comments of the experts and participants, the most frequent observations urged (a) a review the concept of “spiritual beliefs” because it leads to confusion; (b) a reinforcement of the idea of process (dynamic adaptation); (c) a review of the translation of item 10 (diffuse); (d) a review the proportionality of the item valuation scale; and (e) an evaluation of the specific translation suggestions. The direct translators were consulted, and following the author’s criteria, item 10 was modified, and the translation was adjusted following the suggestions provided (e.g., “physical condition” replaced “physical capabilities” and “as I become a senior” replaced “as I grow older”, among others). Thus, the definitive version with which the content validity analysis was performed was obtained, and the results are displayed in Table 1.

**Table 1:** Content validity indices by item and global scores. Alcalá, Madrid, Spain, 2017

	I-CVI*	Pc^†^	κ^‡^	S-CVI^§^
i.1^║^	0,9473	3,6239E-05	0,9473	-
i.2	1	1,9073E-06	1	-
i.3	0,8947	0,0013	0,8945	-
i.4	0,9473	3,62396E-05	0,9473	-
i.5	1	1,90735E-06	1	-
i.6	0,8947	0,0013	0,8945	-
i.7	0,9473	3,62396E-05	0,9473	-
i.8	1	1,90735E-06	1	-
i.9	0,8947	0,0013	0,8945	-
i.10	0,8421	0,0665	0,8308	-
i.11	1	1,90735E-06	1	-
i.12	0,8421	0,0665	0,8308	-
i.13	0,9473	3,62396E-05	0,9473	-
i.14	0,8421	0,0665	0,8308	-
i.15	0,8333	0,1120	0,8122	-
				
Mean	0,92	0,02	0,91	0,92
95% CIs^¶^	0,88 - 0,95	0,00 - 0,04	0,88 - 0,95	0,88 - 0,95
Colombian version	-	-	0,86	0,97

*I-CVI - Item Level Validity Calculation; †Pc -Probability of Chance Agreement; ‡κ - Modified Kappa Coefficient Designating Agreement on Relevance; §S-CVI - Overall Scale Average; ║i.1-15 - Items 1-15; ¶95% CIs - 95% confidence intervals

A sample of 138 participants was recruited. Of these participants, two did not meet the selection criteria (under 20 years of age), and one did not consent to participate. A total of 116 participants completed the questionnaire; of these, 66 agreed to perform the retest, with 65 actually completing it. The sample consisted of 90 women (77.59%) and 26 men (22.41%).The mean age of the women was 39.71 years (95% CIs = 30.81 - 41.26), and that of the men was 43.38 years (95% CIs = 41.83 - 52.28). The remaining descriptive statistics are presented in Table 2.

**Table 2:** Descriptive statistics: Main sociodemographic variables (n=116). Alcalá, Madrid, Spain, 2017

	H*	%	M^†^	%	Total	%
*Marital status*						
Married	11	9.5	28	24.1	39	33.6
Divorced	2	1.7	12	10.3	14	12.1
Common-law marriage	0	0.0	11	9.5	11	9.5
Single	13	11.2	36	31.0	49	42.2
Widow/er	0	0.0	3	2.6	3	2.6
*Education level* ^‡^						
None	0	0.0	0	0.0	0	0.0
Primary	2	1.7	4	3.5	6	5.2
Secondary	5	4.3	10	8.7	15	13.0
University	18	15.7	76	66.1	94	81.7
*Work situation* ^§^						
Unemployed	1	0.9	9	8.0	10	8.8
Student	3	2.7	13	11.5	16	14.2
Retired	1	0.9	2	1.8	3	2.7
Working	21	18.6	62	54.9	83	73.5
Long-term unemployment	0	0.0	1	0.9	1	0.9
*Number of children* ^║^						
0	11	9.6	50	43.9	16	53.5
1	4	3.5	13	11.4	17	14.9
2	9	7.9	17	14.9	26	22.8
3	1	0.9	8	7.0	9	7.9
> 3	1	0.9	0	0.0	1	0.9
*Chronic disease* ^¶^						
Yes	7	6.1	28	24.3	35	30.4
No	19	16.5	61	53.0	80	69.6
*Hospitalisations*						
Yes	2	1.7	16	13.8	18	15.5
No	24	20.7	74	63.8	98	84.5
Mean age**	43.3	(41.83-52.28)	39.7	(30.81-41.26)	40.5	(38.11-42.95)

*H - Men; †M - Women; ‡1 missing value; ║2 missing values; §3 missing values; ¶1 missing value; **95% CIs are indicated

**Table 3:** Goodness-of-fit indices for the resulting factor models. Alcalá, Madrid, Spain, 2017

	Four factors*	Three factors^†^	Two factors^‡^	One factor^§^
χ^2║^ *p* ^¶^ df**	111,537 0.023 84	111,673 0.038 87	117,577 0.022 89	152,377 <0.001 90
GFI^††^	0,896	0,896	0.891	0.854
AGFI^‡‡^	0.852	0.857	0.853	0.806
RMSEA^§§^	0.053	0.049	0.053	0.077
NFI^║║^	0.707	0.707	0.691	0.600
NNFI^¶¶^	0.875	0,892	0.878	0.736
CFI***	0.900	0.910	0,896	0,774
SRMR^†††^	0.069	0.068	0.071	0.837

*Four-factor model (F1: items 3,6,8, and 9; F2: 11,12, and 5; F3: 1,2,4,7, and 14; F4: 10,13, and15); †Three-factor model (F1: 3,6,8, and 9; F2: 11 and 12; F3: 1,2,4,5,7,10,13,14, and 15); ‡Two-factor model (F1: 3,6,8, and 9; F2: 1,2,4,5,7,10,11,12,13,14, and 15); §One-factor model (F1: 1,2,3,4,5,6,7,8,9,10,11,12,13,14, and 15); ║χ*2* - Chi- square; ¶*p* - statistical significance for χ*2*; **df - degrees of freedom

The correlations obtained between the pairs of scales, all of them, presented significant values (*p* < 0.001). The greatest correlation was between the FACIT-Sp-Non-Illness and PWB scales, with *r* = 0.70. The STS was moderately and positively correlated with these previous scales (*r* = 0.68 and 0.63, respectively). The STS showed a higher mean score for n = 65 (this sample includes the 65 participants who answered the test and retest portions), with a mean of 0.86 (0.65 for FACIT-Sp-Non-Illness and PWB scales).

**Figure 1:**
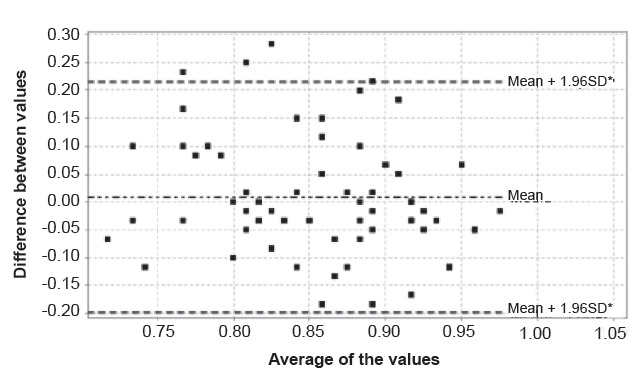
Bland-Altman plot of the TRT^†^ agreement analysis

**Figure 2:**
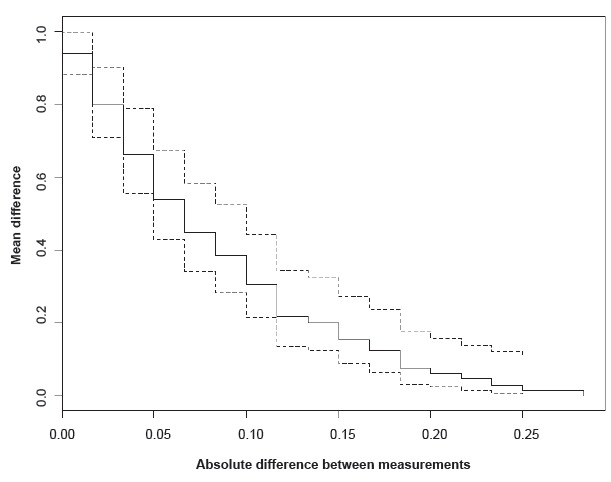
Kaplan-Meier curve of the TRT* agreement analysis

Regarding reliability, when the TRT scores were considered the object and the researcher was considered the observer, the ICC was 0.278 (*p* = 0.0972, 95%CIs = -0.183 - 0.56). The inter-observer reliability was 0.932 (*p* < 0.001, 95% CIs = 0.891 - 0.963). Student’s t-test for paired samples yielded a *p-*value of 0.533, with an estimated value of 0.008 (95% CIs = -0.017 - 0.034). A graphic explanation of the TRT concordance was obtained using the Bland-Altman and Kaplan-Meier methods, represented in Figures 1 and 2. The former shows that all of the scores are within the 95% CIs except four that exhibit high TRT differences. The latter shows not only that the differences are within the 95% CIs but also that the probability of discordance decreases as the TRT difference increases.

*SD - Standard Deviation; †TRT - Test-retest

*TRT - Test-retest

Bartlett’s sphericity test revealed a result of χ^*2*^ = 359.625, df = 1,050, and *p* < 0.001, and the Kaiser-Meyer-Olkin metric, with a result of 0.720, confirmed the relevance of a factorial analysis. In the EFA, several extractions were made because models with one, two, three, and four factors were possible (using eigen values> 1 and factorial loadings > 0.30). However, their *p*-values (H_0_: *x* factors are sufficient) were only significant in the one-factor model (*p* = 0.0002) and the two-factor model, although the significance threshold was slightly exceeded (*p* = 0.0545). When comparing the factorial loads of the original matrix with those of the varimax and promax rotations, items 3, 6, 8, and 9 constituted an independent factor in all the models, as did items 11 and 12. The compositions of the models are shown in Tables 4 and 5, which also display the eigen values of the four-factor model without rotation and with the varimax and promax rotations. The cumulative explained variance decreased with the number of factors (41.4% with four factors to 21% with one factor), which is unlike the chi-square parameter (χ^*2*^ ) that increased from 55.83 with four factors to 143.69 with one factor. The individual fit of the items to each factor (*R* ^2^) revealed a better global fit for the three-factor model, followed by the two-, four-, and one-factor models in that order. At least one factor was negatively correlated in all the models, with progressively higher values as the number of factors extracted decreased (-0.26 in the four-factor model to -0.46 in the two-factor model). The CFA added goodness-of-fit indices to all of the models (Table 4). Cronbach’s α TRT coefficient provided the following results: α_*t*_ = 0.772 (0.785 standardised) and α_*rt*_ = 0.833 (0.844 standardised). Variation in α was observed when eliminating each item. When eliminating item 12 in the test, α_*t*_ increased to 0.783 (0.783), whereas without this item, it remained below the initial value. The same issue occurred in the retest, increasing α_*rt*_ to 0.840 (0.841).

The following feasibility results were obtained: 4% of the total participants commented on the scale, primarily focusing on the concept of “spiritual beliefs” and the disproportionality of the item valuation scale. The average completion time was 13.090 minutes (*p*< 0.001, 95% CIs = 11.771 - 14.410) including the complete filling process. Six observations were missing on the three scales in the test phase (0.09%), and five were missing in the retest phase (0.07%). Regarding the STS, one was missing in the test (0.01%) and retest (0.1%) phases each. Of the 138 people who accessed the platform, 22 (15.94%) did not complete the questionnaires or the other mandatory information.

## Discussion

Some of the experts in this study commented on the debate concerning the translation of the syntactic construction of items that required an adjustment for that reason (i.e., items 2, 4, 5, and 15). However, keeping all the items in gerund form implied that the person questioned does not see him or herself in the present moment but outside the space-time margin. Reed developed the items to avoid biases with healthy people and measure the ability to find well-being through cognitive, creative, social, spiritual, and introspective resources. According to her theory, self-transcendence is a *multidimensional* fluctuation of personal limits, independent of the state of health. The person can find him or herself before, during, or after one or several adaptive processes. This scale attempts to measure the person’s viewpoint at that moment in life and not during a hypothetical moment of reflection defined as an abstraction. Therefore, a balance between transcendence and immanence was needed to naturally address a process. For example, in the case of a person who is bedridden and in response to item 1, *having* hobbies is adequate but does not face reality; however, “*I have* hobbies or interests that I enjoy” indicates that, whether actively or passively (nursing care intervenes here), the person really enjoys certain hobbies. This change creates a significant difference when scoring the scale. It is not only a process of abstraction but also a cognitive, experience, and multidimensional adaptation.

The experts and participants also cited the need to clarify “spiritual beliefs” (item 12). We considered that discriminating between spirituality and religiosity is necessary in light of the religious situation in Spain. According to the last barometer of the Centre for Sociological Research (CIS), July 2017^21^, 68.8% of respondents (n = 2,490) consider themselves Catholic; however, 58.9% of believers (n = 1,771) do not practice. However, the adaptation of the scale does not pretend to conform to a confessional situation but to the intention of the author. The creation of instruments to measure spirituality in the field of health has created controversy, and the tendency for years has been to separate both concepts^17^. Spirituality has expanded its dimensions, relating to transcendence as well as the search for purpose and meaning in life, something that is individual and born of the person. On the other hand, religion is considered participation in dogmatic, institutionalised, and sanctioning beliefs as well as in activities of groups with a particular faith^17^. Therefore, the use of “spiritual beliefs” was considered justified, and the problem is one of interpretation and not a lack of definition. This issue continues to cause discrepancies at a social level and is not always well received; specifically, it caused certain misplaced comments during the promotion of this study, which shows that this issue cannot be considered completely assimilated.

The disproportionate number of items on the valuation scale was discussed with the author, who answered that her intention was to anchor the values in an equidistant way and to allow for subjective appraisals when assessing. The author approved the scale proposed in the current study and proposed an alternative option of indicating only the extremes (not at all, a lot) with two intermediate options without value. The translators decided to respect the original structure of the author for psychometric reasons. If a common interpretation exists, then it is understood that the results will be equally proportional.

The resulting scale scored well. Although Cronbach’s α was not excellent, it was within the range of values of the versions cited in the introduction (0.77 - 0.83). The conflict with item 12 (spiritual beliefs) might be explained by the difficulty of discriminating spirituality and religion in a single item. In any case, the increases in α_*t*_ and α_*rt*_ when eliminating this item is high enough to modify the degree of internal consistency (difference of means = -0.009; *p* = 0.139, 95% CIs = -0.034 - 0.016). Comparing these results with the items that contain this concept in the FACIT-Sp-Non-Illness scale revealed a mean difference of -0.006 (*p* = 0.106, 95% CIs = -0.015 - 0.001).The results of both scales were in same direction, which shows that the relationship between this item and the scale is not anomalous. Regarding inter-observer and intra-observer reliabilities, the first coefficient was 0.932, which indicates satisfactory agreement between participants and that the variability is due to the differences between them. The latter ICC was 0.278, which can be interpreted as (a) low agreement between the TRT scores, (b) the instrument not measuring reliably, or (c) this agreement being partially due to chance. As such, the limitation in the sample size must be considered. When interpreting ICC values, any classification is subjective^18^. In this case, the STS might not be an accurate instrument, and these differences cannot be evaluated in a sensitive way. We did not find any reference to grade the scores of the scale to investigate the degree of deviation; therefore, an alternative gradation was developed by dividing the maximum score by 10 (base 10). The result was six points; therefore, a difference of one degree equals six points (0.10 expressed in ratio). The standard deviation of the differences in the means, indicated in the Bland-Altman plot, is 0.105 (approximately sixpoints), and the confidence ranges are 0.20 (12 points). Therefore, the deviation of the scores is not high (less than two degrees or less than 20%), and they range within the confidence intervals. This finding is also shown in the Kaplan-Meier curve, which indicates that the probability of the difference being one degree (0.10 or six points) is approximately 0.3 (30%). Furthermore, by increasing that difference (> 0.10), the probability of discordance becomes progressively smaller until reaching zero. The apparent validity was not remarkable, and the overall significance of the instrument, represented by S-CVI, was 0.92 (95% CIs = 0.88 - 0.95), which indicates high validity. The correlations with the reference scales showed highly significant *p*-values for a moderate correlation; importantly, however, the sample size is not large, and the three scales share factors but do not measure the same concepts. The fit measures of the CFA determined better results for the three-factor model that did not coincide with the theoretical basis that states that the scale should be one-dimensional. Other studies have also observed differences: the Korean version^3^ revealed four factors; the Persian version^5^ showed two factors and a Norwegian study that investigated the multifactorial nature of the scale^6^ showed that the best fitting model was two factors. Our case results also suggest that two main factors are revealed: the content of items 11 and 12 refer to a transpersonal dimension, whereas items 3, 6, 8, and 9 clearly refer to a social dimension. The remaining items comprise a block that mixes intrapersonal and temporal facets. Item 1, which initially loaded on the same factor as items 11 and 12, was forced to move to the intrapersonal dimension factor to adapt the model better to the theory, providing better results in the CFA. Therefore, the modification was maintained. Although it does not coincide with the four theoretical dimensions, the three-factor model was more stable.

The final sample size was affected by a time limitation, which places the generalisation of the results at risk, even though they are statistically significant. Other limitations are inherent to this type of study, including (a) the methodological design itself (i.e., the use of the Internet as a means to complete questionnaires favours selection bias); (b) numerous participants were university students because the promotional environment was close to the researcher (possible selection bias); (c) the difficulty of establishing an adjusted TRT interval (possible memory bias);and (d) not knowing the causes of the non-completion of questionnaires or retesting (possible information bias). In addition, few volunteers were available to select as translators and experts. Although the methodology suggested that bilingual and bicultural translators be used for both phases^12^, this was only possible for the reverse translation; nevertheless, this criterion is only recommended and not considered essential.

The Colombian reference version^9^ presented similar results, with differences in the factorial structure (a single factor explained 36.18% of the variance).

## Conclusions

The results of this study justify the validity and applicability of this scale in Spain. Although this line of research should be continued with appropriate adjustments, we conclude that a starting point already exists, which implies that the research objective (to elaborate the Spanish version of the STS) was fulfilled.

Compared with the Colombian version, certain variations imply that a significant change was made; at the same time, a critical analysis was necessary for this cross-cultural adaptation. Without taking into account these differences, this new version provides the following improvements: (a) more natural and fluid writing, (b) greater syntactic correction, (c) the use of inclusive language, (d) an extended target population, (e) a greater conceptual definition, and (f) an alternative factor model. A dynamic equilibrium must be maintained to enable the improvement of its psychometric properties, which remain relevant.
